# An Efficient Approach in Analysis of DNA Base Calling Using Neural Fuzzy Model

**DOI:** 10.1155/2017/3686025

**Published:** 2017-01-31

**Authors:** Safa A. Hameed, Raed I. Hamed

**Affiliations:** ^1^College of Computer Science and Information Technology, University of Anbar, Al-Anbar, Iraq; ^2^College of Science and Technology, University of Human Development, Sulaymaniyah, Iraq

## Abstract

This paper presented the issues of true representation and a reliable measure for analyzing the DNA base calling is provided. The method implemented dealt with the data set quality in analyzing DNA sequencing, it is investigating solution of the problem of using Neurofuzzy techniques for predicting the confidence value for each base in DNA base calling regarding collecting the data for each base in DNA, and the simulation model of designing the ANFIS contains three subsystems and main system; obtain the three features from the subsystems and in the main system and use the three features to predict the confidence value for each base. This is achieving effective results with high performance in employment.

## 1. Introduction

A neural fuzzy theory approach is considered; it presented the combined advantages of both fuzzy logic and neural networks [1]. Fuzzy logic inferencing could be implemented in production networks by manually setting the offsets. This procedure, however, receives criticism, since there is a feeling that neural networks should include training [2, 3]. The combining of neural networks and fuzzy logic allows for the possibility of solving adjustment problems and the design constraints which are found in fuzzy logic [4, 5]. And more importantly the Neurofuzzy is efficient to apply in biosystem and in genomics. The study in genomics has been increasingly important to biologists and workers in this field, permitting them to continue analysis of the pattern of thousands of genes in a single experiment [6]; the Neurofuzzy technique can be used as a method to identify the changes of the statistics to determine if some “agent” is capable of predicting (and thus recognizing) [7] and in the functional analysis of gene expression data from microarray experiments [8]; and it has been applied in DNA base calling and demonstrated that differentiation in contextual sequencing trace data peak heights actively encodes the new information that can be used in base calling and confidence estimation. By using the Neurofuzzy classifier, it is able to decode much of the hidden contextual information in two fuzzy rules per base and partially discover its main behavior [9]. In this research, we use the Neurofuzzy approach to predict the confidence value in each base in DNA base calling; we have three subsystems to determine the three features of the base and use them as the input in the main confidence Neurofuzzy system.

## 2. Literature View

There are several attempts to use the Neurofuzzy technique in more applications, Neurofuzzy approaches mix between fuzzy inference systems (FISs) and neural networks (NNs) and combine the advantages of both of them, because of neurofuzzy approach's successful practical technology in many areas. The neural fuzzy (NF) systems take a place in the field of genomics; the paper [10] suggests using different Neurofuzzy tools able to recognize specific sequences; the paper [8] proposed the application of Neurofuzzy techniques for the functional analysis for gene expression data depending on microarray experiments, by a way of combining declared and hidden knowledge in functional interpretation and analysis of gene expression data is suggested, and [7] explains the processing in the prediction of the gene structure by a new method and tools, which involve the sequence of distances between bases and Neurofuzzy predictors.

The paper [11] presented an algorithm that has been created for the determination of nucleotide sequences from information delivered in fluorescence-based mechanized DNA sequencing instruments utilizing the four-color methodology. This algorithm exploits object-oriented programming strategies for modularity and extensibility. Confidence values are given on the base calls as an estimate of accuracy. The algorithm iteratively utilizes confidence determinations from a few distinct modules. The paper [12] depicts a neural network model for photometric signal molding amid crude information securing with a mechanized DNA sequencer. This network bolsters online extraction and evaluation of instructive arrays of oligomer partitions and this will make a table giving you values of the real time base calling. In [13] another base calling algorithm that is proposed to be autonomous of a specific sequencing innovation has been produced and appeared to be viable with information from the Applied Biosystems 373 sequencing framework. This algorithm makes utilization of a nonlinear deconvolution filter recognizing likely oligomer events and a diagram theoretic editing system to search the well subset by the way of comparing with right sequence.

The important part of this work is to determine the confidence value in development way of using the Phred base calling. By employing an algorithm on a huge data set Phred was able to make a model (a lookup table). The input space of the model consists of trace data features such as the peak spacing, uncalled/called ratio, and peak resolution. The output space is the resulting quality value. Creating a confidence value for the base calls, in this procedure of traces and quality values using Consed [15] explained that the quality assignments obtained using the system portrayed as a piece of methods contrast truly well with quality judgments by a human analyst of the data. In [16], a fuzzy Petri net (FPN) technique to modeling fuzzy rule-based reasoning is proposed to determine the confidence values for bases called in DNA sequencing. This contains the conversion of fuzzy rules into FPN, side by side with their reasoning. The paper [17] illustrates the technique developed which uses the fuzzy logic. This technique used to provide the confidence values of bases called. Three variables are calculated during the base calling process which are participating in the fuzzy system. The technique progress results in the software (Trace Tools) that was created based on the preprocessing and base calling algorithms. With this information about the three variables we use it as the inputs after collecting it in our subsystems Neurofuzzy model in this paper and use it in the main Neurofuzzy system, in order to determine the confidence value in DNA sequencing, by utilizing and gathering the information about the bases in DNA sequencing, and we design the model by the optimized method to predict the value of the analysis.

## 3. The Neural Fuzzy Technique Based on DNA Base Calling

The neural fuzzy (NF) systems can achieve a higher accuracy within a relatively short training time comparing with neural networks and the difficulty of choice and building of membership functions in the fuzzy logic of a given problem. Unlike other applications, the neural fuzzy (NF) techniques are more transparent models and efficient to implement.

The Neurofuzzy classifier is used on the other side of implementations, which is able to decode much of the hidden contextual information in two fuzzy rules per base and partially discover its underlying behavior [9]. Neural networks have been applied with good results to functional genomics problems because of their capabilities to deal with complexity, uncertainty, and noisy or corrupted data. Fuzzy technique can be suitable for analyzing bioinformatics data as it allows the integration; the advantages of the neural network over fuzzy systems are learning and adaptation capabilities, whereas the advantages of fuzzy systems are the human understandable form of knowledge representation. The Neural networks use an implicit method of knowledge performance, while fuzzy and Neurofuzzy systems provide the knowledge in explicit forms, such as rules [14]. In our model, we analyze the DNA base calling regarding collecting data (for more information see [17]) to predict the confidence value for each base by using the Neurofuzzy system.

## 4. The Simulation of the Neurofuzzy Method in DNA Base Calling

### 4.1. Background

**Figure 1:**
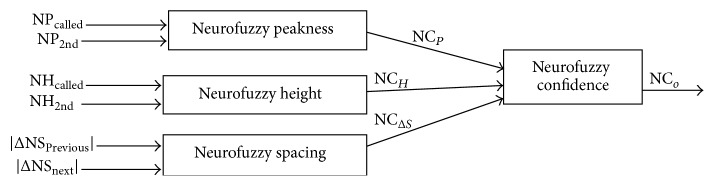
Model overview of ANFIS main confidence system.

A Neurofuzzy technique is implemented by the designing which is suitable for determining the confidence value of base calling. This designing includes three subsystems to determine the three features regarding the collected data at each base (for more information about the data collection see [17]) and the main system that inputs the three features obtained and determine the confidence value for each base. We generate the ANFIS for each and obtain the value through the training and testing of the system by loading the data set file of samples; see Figure 1. We select the suitable parameters from the Neurofuzzy technique that gives an efficient result for each system.

### 4.2. The Method

We use the Neurofuzzy technique, which is the optimal method to measure incorrect analysis and editing process to make it much easier and faster with the results and give the credible and true representation of the values. The Neurofuzzy technique here very well establishes the method and has a tremendous potentiality to give results with high accuracy ratio and the efficiency in dealing with DNA sequencing; this approach is proposed regarding collecting information at the base; for more information see [17].

This designing includes the three subsystems to determine the three features (peakness, height, and spacing) and the main system that inputs the three features obtained, and we determine the confidence value for each base in DNA base calling by using the MATLAB tool; see Figure 1.

In this system we use the four files of data set that contain 500 samples for each, and each file is divided into two parts: training set and testing set through implementing the method; in the training set, we use the data set of about 350 samples, whereas, in the testing set, we use the data set of about 150 samples. In the first, in the training step, we load the file training set of data sample for DNA base calling, and in the fuzzy inference system two inputs and one output for each system in the three subsystems are generated to obtain the three features and three inputs and one output in the main system in order to obtain the confidence value for each base. We attempt several processes in ANFIS subsystems and ANFIS main system and choose the most suitable one with less average testing error.

**Figure 2:**
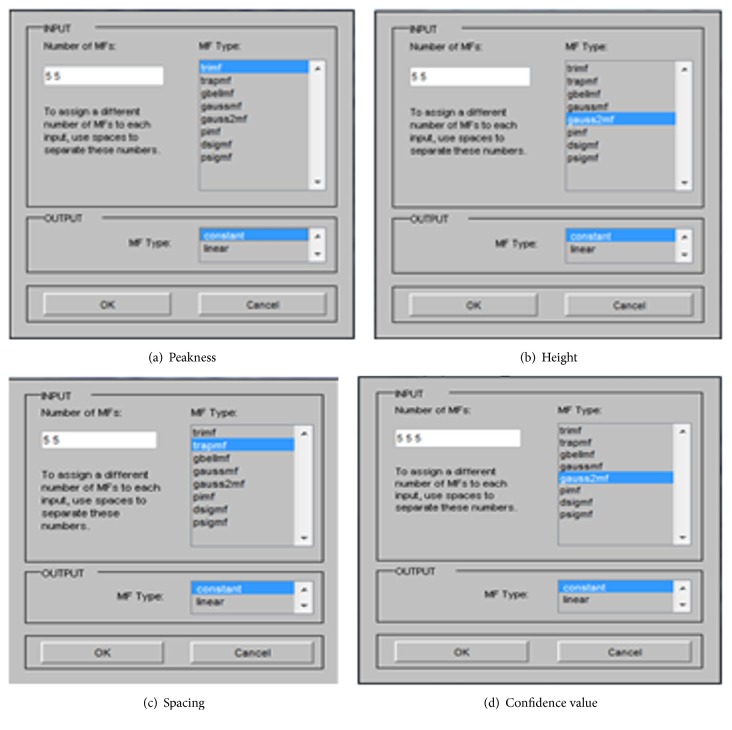
(a, b, c) The membership function of each feature in the subsystem, and (d) is the membership function for the confidence value of the main system.

According to the membership function of this system, we generate five MF for each input in each system, in the subsystem design, in the peakness, we generate the triangular MF, in the height, we generate the gauss 2 MF, and, in the spacing, we generate the trapezoidal MF; in the main confidence system, we generate the gauus 2 MF, with constant output for each of the three subsystems and the main one; see Figure 2.

**Figure 3:**
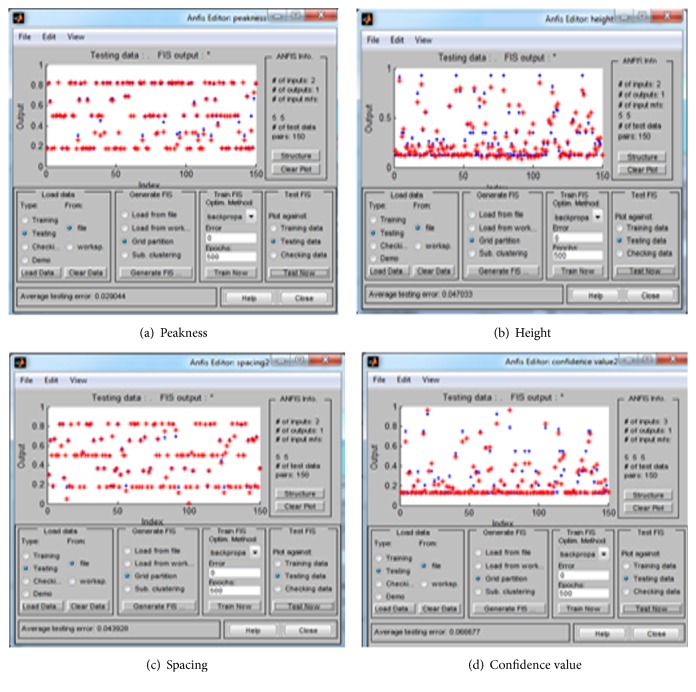
(a, b, c) The ANFIS testing results for each feature in the subsystem, and (d) is the ANFIS testing results in the confidence value in the main system.

Then perform training with backpropagation in neural fuzzy system with 500 epochs, as well as testing the system by loading the file testing set. Through this method, we select the option that reaches the result in very high accuracy; see Figure 3. Explain the training and testing for the three features in the subsystems and for the main system.

### 4.3. Result and the Discussion

By using the Neurofuzzy technique, we obtained the results with high performance through building the optimal designing, through training and testing the system; the if-then rules are generated automatically in suitable way that helps us to give the result with reaching success in the correct analysis depending on data set that is loaded in the system; when the system is tested and the rules are generated the structure of the Neurofuzzy system is applied, and we can use the rule view to get the results for each system. To illustrate our method a section of a DNA sequence which includes six bases (ATCTCG) is used.  Table 1 illustrates the input data, and we use it in the three subsystems to get the three features (peakness, height, and spacing).

**Figure 4:**
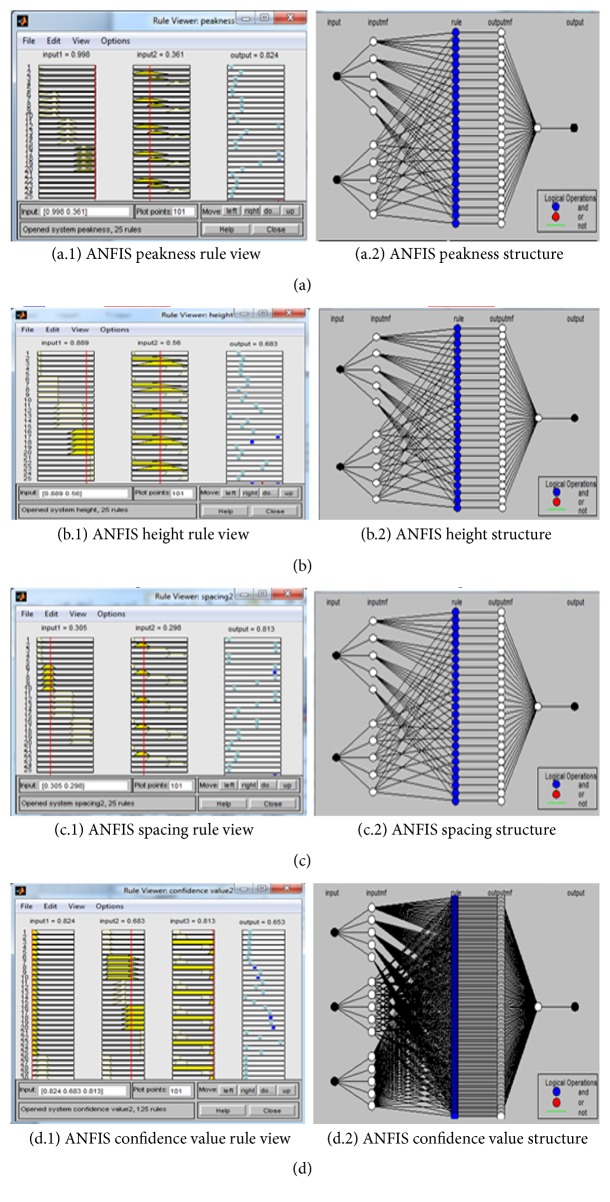
(a, b, c) The ANFIS rule view and the structure for the three fuzzy subsystems and (d) for the main fuzzy system.

Figure 4(a.2) shows the ANFIS structure of the peakness subsystem according to the generation of the if-then rules and the FIS. Figure 4(a.1) shows the two inputs for each base in the section of the DNA sequencing: NP_called_, NP_2nd_ and the result in peakness (NC_*P*_). For example, for base A, NP_called_ is 0.998, while NP_2nd_ is 0.361. This provides a peakness value NC_*P*_ of 0.824 for that base, and so on. Figure 4(b.2) shows the ANFIS structure of the height subsystem according to the generation of the if-then rules and the FIS. Figure 4(b.1) shows the two inputs for each base in the section of DNA sequencing: NH_called_, NH_2nd_ and the result in height (NC_*H*_). For example, for base A, NH_called_ is 0.889, while NH_2nd_ is 0.560. This provides a height value NC_*H*_ of 0.683 for that base, and so on. Figure 4(c.2) shows the ANFIS structure of the spacing subsystem according to the generation of the if-then rules and the FIS. Figure 4(c.1) shows the two inputs for each base in the section of DNA sequencing: ΔNS_next_, ΔNS_Previous_ and the result in spacing (NC_Δ*S*_). For example, for base A, ΔNS_next_ is 0.305, while ΔNS_Previous_ is 0.298. This provides a spacing value NC_Δ*S*_ of 0.813 for that base, and so on.

From the above, we obtained the value of the three features (peakness, height, and spacing), and now we use these values as the three inputs in the main ANFIS confidence system to determine the confidence value for each base in DNA base calling. Figure 4(d.2) shows the ANFIS structure of the confidence value system according to the generated if-then rules and the FIS. Figure 4(d.2) shows the three inputs for each base in the section of DNA sequencing (peakness, height, and spacing) and giving the confidence value output. The result in Table 2 illustrates the corresponding values for the Neurofuzzy confidence. For example, for base A, the peakness is 0.824, the height is 0.683, and the spacing is 0.813. This provides a confidence value NC_*o*_ of 0.653 for that base, and so on.

## 5. Conclusion

The main idea of this paper is using an efficient technique implemented in DNA base calling in order to determine the confidence value for each base; the combination of neural network and fuzzy logic has overcome the difficulties through building the membership function in fuzzy logic and the constraints in implementing the neural network, which gives efficient results with high performance, by designing the ANFIS for each of the three subsystems to obtain the three features and the main system to predict the confidence value for each base.

## Figures and Tables

**Table 1:** The input data value for each base.

Features	Input data	The bases of the sequences					
		A	T	C	T	C	G
Peakness	NP_called_	0.998	0.999	0.794	0.999	0.930	0.999
	NP_2nd_	0.361	0.478	0.838	0.721	0.665	0.618

Height	NH_called_	0.889	0.991	0.644	0.954	0.696	0.952
	NH_2nd_	0.560	0.421	0.604	0.606	0.531	0.485

Spacing	ΔNS_next_	0.305	0.305	0.281	0.286	0.302	0.274
	ΔNS_Previous_	0.298	0.305	0.305	0.281	0.286	0.302

**Table 2:** Confidence value for bases called.

Input data	Bases					
	A	T	C	T	C	G
Peakness	0.824	0.825	0.499	0.499	0.612	0.766
Height	0.683	0.767	0.230	0.692	0.390	0.741
Spacing	0.813	0.813	0.813	0.812	0.813	0.813
Confidence value NC_*o*_	**0.653**	**0.747**	**0.205**	**0.661**	**0.364**	**0.716**
